# Potent anti-tumor activity of telomerase-dependent and HSV-TK armed oncolytic adenovirus for non-small cell lung cancer in vitro and in vivo

**DOI:** 10.1186/1756-9966-29-52

**Published:** 2010-05-20

**Authors:** Ju-Feng Zhang, Fang Wei, Hui-Ping Wang, Hui-Ming Li, Wei Qiu, Peng-Kang Ren, Xia-Fang Chen, Qian Huang

**Affiliations:** 1 Central Experimental Laboratory, First People's Hospital, Shanghai Jiaotong University, 85 Wujin Road, Shanghai 200080, PR China; 2 School of Life Science, Guangdong Pharmaceutical University, Guangzhou 510006, PR China

## Abstract

**Background:**

Non-small cell lung cancer (NSCLC) is the leading cause of cancer related mortality, any improvements in therapeutic strategies are urgently required. In this study we generated a novel 'suicide gene' armed oncolytic adenoviral vector and investigated its antitumor effect both in vitro and in vivo.

**Methods:**

Since the up-regulated expression of human telomerase reverse transcriptase (hTERT) is a hallmark of alltypes of NSCLC, we chose hTERT promoter to transcriptionally control E1A gene expression to obtain adenoviral replication in NSCLC. In order to further enhance anti-tumor effect of this oncolytic adenoviral vector, we inserted a 'suicide gene' i.e. Herpes Simplex Virus Thymidine Kinase (HSV-TK) into oncolytic adenoviral vector to engineer a novel armed oncolytic adenoviral vector 'Ad.hTERT-E1A-TK'.

**Results:**

Ad.hTERT-E1A-TK efficiently killed different types of tumor cells including two types of NSCLC cells *in vitro*, causing no damage to normal primary fibroblasts. Furthermore, Ad.hTERT-E1A-TK infection combined with administration of prodrug gancyclovir (GCV) resulted in more potent cytotoxicity on NSCLC cells, and synergistically suppressed human NSCLC tumor growth in nude mice.

**Conclusion:**

The results from this study showed that Ad.hTERT-E1A-TK/GCV could be a potent but safe anti-tumor strategy for NSCLC biotherapy.

## Background

Lung cancer is one of the most common malignant neoplasm diseases in which non-small cell lung cancer (NSCLC) constitutes 80%-85% of all lung cancers [1]. Due to the lack of early diagnostic methods, most of NSCLC cases are diagnosed at the late phase and patients usually lose the opportunity of surgical treatment. Despite the fact that chemotherapy and radiotherapy provides many options to treat NSCLC, a survival plateau has been reached and its mortality is still in the first place in cancer patients [2,3]. Therefore it is urgent to explore other treatment strategies. Molecule targeting therapy represents a rapidly growing cancer treatment strategy and several drugs have been proven effective in many preclinical and clinical setting [4,5]. Suicide gene therapy possesses the advantage of molecule targeting strategies because the suicide gene functions in the transformed tumor cells and then selectively kills transformed tumor cells and their surrounding cells via bystander effects. In some extent, the suicide gene therapy could overcome the systemic toxicity of conventional chemotherapy. Herpes Simplex Virus Thymidine Kinase/gancyclovir (HSV-TK/GCV) is one of the most frequently utilized forms of suicide gene therapy. HSV-TK can catalyze GCV into monophosphorylated GCV (GCV-MP) that will then be converted into toxic gancyclovir triphosphate (GCV-TP) by other cellular kinases and thereafter cause cell growth inhibition or initiates cell death. According to previous studies NSCLC is a good target for HSV-TK gene therapy [6].

How to efficiently and selectively deliver HSV-TK gene into tumor cells? It has been reported that the non-replicative adenoviruses were able to infect and mediated gene transfer into NSCLC [7]. The replication-competent adenoviruses, also called oncolytic adenoviruses, are thereby a natural extension from the success of non-replicative adenoviruses mediated gene delivery. The advantage of using the replication-competent adenoviruses for therapeutic gene delivery is that it can selectively replicate and spread in malignant tumor tissues, and finally lead to remarkably increased therapeutic gene expression in tumor cells accompanying adenoviral replication and spread. The current strategy to generate tumor-selective replication-competent adenovirus is to replace the adenovirus E1 gene promoter with other tumor or tissue-specific promoter [8,9]. Since human telomerase reverse transcriptase (hTERT) is over-expressed in all types of NSCLC, but is inactivated in normal cells [10], in the present study, we chose the hTERT promoter to drive adenoviral E1A gene expression and generated a tumor-selective replication-competent adenoviral vector. In order to further enhance therapeutic efficacy, we inserted a constitutive expression HSV-TK gene into this vector to develop a novel armed oncolytic adenovirus (Ad.hTERT-E1A-TK). We subsequently evaluated whether Ad.hTERT-E1A-TK could preferentially replicate in NSCLC and HSV-TK/GCV system could effectively kill NSCLC both in vitro and in vivo.

## Materials and methods

### Cells and cell culture

HEK293 (human embryonic kidney 293) cells were purchased from Invitrogen (San Diego, CA, USA). NCIH460 (human large cell lung cancer), A549 (human lung adenocarcinoma), SW1990 (human pancreas cancer), Hela (human cervical carcinoma), and SMMC-7721 (human hepatoma) were obtained from the Cells Bank of the Chinese Academy of Science (Shanghai, China). Primary human dermal fibroblasts were provided by our laboratory, and which was derived from bioptic tissue for dermatoplasty (a written informed consent was obtained from patients). Cells were cultured in DMEM or RPMI 1640 medium supplemented with 10% fetal bovine serum (FBS).All of the tumor cells had activated telomerase activities, while primary human dermal fibroblasts showed lower telomerase activity according to our previous study.

### Adenoviral vectors

Ad.hTERT-E1A-TK is an oncolytic adenoviral vector that is able to replicate in hTERT activated tumor cells and carries a constitutive expression HSV-TK-HAtag cassette. The construction of Ad.hTERT-E1A-TK has been described previously [11]. Ad.GFP is a replication-defective Ad that lacks adenoviral E1A gene and expresses the green fluorescent protein gene (GFP). Ad.null is also a replication-defective Ad but expresses no extraneous gene. The dl309 is a wild-type Ad that contains intact adenoviral E1 gene. Ad.hTERT-E1A and Ad.hTERT-E1A-CD had similar structure with Ad.hTERT-E1A-TK and were used as positive control for oncolytic adenovirus. The former contains no therapeutic gene while the later has Escherichia colicytosine deaminase gene (E coli CD) instead of Herpes Simplex Virus Thymidine Kinase (HSV-TK). Purified, high titer viral stocks were generated according to the protocol described by Huang et al [12]. The schematic diagram of Ad.hTERT-E1A-CD or Ad.hTERT-E1A-TK was shown in Additional file 1.

### Western blot analysis

The adenoviral HSV-TK and E1A expression was confirmed by Western blot as described below. The cells were plated in 6-well plates and infected with the Ad.hTERT-E1A-TK at a multiplicity of infection (MOI) of 10 plaque forming units (PFU) per cell. Cells were harvested and lysed 48 h later after infection in SDS sample buffer (containing 10 mM β-mercaptoethanol, 100 mM Tris-CL [pH 6.8], 2% SDS, and 0.1% bromophenol blue). Protein samples were separated by 10% SDS polyacrylamide gel and transferred to a polyvinylidene difluoride membrane (Amersham, Buckinghamshire, UK). The blots were probed with anti-HA (Sigma, St. Louis, MO, USA) monoclonal antibody which detected HSV-TK and anti-Ad2 E1A (Santa Cruz Biotechnology, Santa Cruz, CA, USA) polyclonal antibody, followed by a secondary horseradish peroxidase-conjugated antibody. The antigen-antibody complexes were visualized using the enhanced chemiluminescence kit (Roche, New York, NY, USA) as recommended by the manufacturer.

### Cytopathic effect assays

The cytopathic effect (CPE) was determined by three different methods. At first, tumor cells such as NCIH460, SW1990, SMMC-7721 and Hela were plated into 24-well plates and either infected with different dose of Ad.hTERT-E1A-TK, Ad.hTERT-E1A-CD, dl309, Ad.GFP or treated with prodrug gancyclovir (GCV) or 5-fluorocytosine (5-FC) or untreated on the next day respectively. Five days later the plates were stained with crystal violet and the remaining living cells were determined by intensity of blue color. The 2^nd ^method was Cell Counting Kit-8 assay (CCK-8, Dojindo Molecular Technologies Inc., Gaithersburg, MD, USA) which could quantitatively determine living cells by measuring optic intensity. The tumor cells, NCIH460, A549 and Hela grown in 96-well plates were treated with 10 MOI of Ad.hTERT-E1A-TK, Ad.hTERT-E1A-TK plus GCV or GCV alone. Five days later the remaining living cells were determined by CCK-8 assay. The cytopathic effect was also observed by microscopy for morphologic changes. NCIH460 cells and primary human fibroblasts were plated into 6-well plates and infected with 10 MOI of Ad.hTERT-E1A-TK, dl309, or Ad.GFP respectively on the next day. CPE was monitored and photographed by light microscopy at the different time points.

### Viral replication

To determine viral progeny production, NCIH460 cells (4 × 10^5^cells/well) and primary fibroblasts (4 × 10^5^cells/well) were plated into 6-well plates and infected with Ad.hTERT-E1A-TK at 10 MOI for 4 h. The medium containing extra virus was removed and the cells were washed once with PBS and cultured with fresh mediun. 24 h and 5 days later after infection, the cells were collected and lysed by three rounds of freezing and thawing, and then centrifuged to collect the supernatant. The adenoviral particles in the infected tumor cells or fibroblasts supernatant were determined by plaque assay in HEK293 cells.

### Animal experiments

Specific pathogen-free male athymic BALB/c nude mice, 4-6 weeks old (20-30 g), were obtained from the Institute of Animal Center (Chinese Academy of Sciences, Shanghai, China). Mice were housed five per cage and allowed free access to food and water. All animal procedures were performed according to principles of laboratory animal care (NIH publication No. 85-23, revised 1985) and the current Chinese regulations and standards on the use of laboratory animals. For tumor cell implantation, NCIH460 cells (5 × 10^6^) were subcutaneously injected into the right dorsal lumbar region in 100 μl of phosphate buffered saline (PBS). When the tumors grew up to approximately 100 mm^3^, the animals were randomly divided into 4 groups i.e. Ad.null, Ad.hTERT-E1A-TK, Ad.hTERT-E1A-TK plus GCV and PBS plus GCV, and each group contained at least 7 animals. About 1 × 10^9 ^PFU of Ad.null orAd.hTERT-E1A-TK in 100 μl PBS or 100 μl PBS alone were injected into tumors respectively. On the 3rd day post virus injection, GCV (100 mg/kg/day) was intraperitoneally administered for 14 consecutive days. The tumor growth was assessed by measuring bi-dimensional diameters twice a week with calipers. The tumor volumes (V) were calculated according to the formula V = 1/2ab^2 ^(a represents the largest diameter and b represents the smallest diameter). All animals were killed 4 weeks later after treatment and then the tumors were removed and weighed.

### Histopathologic examination of tumors

The resected tumors were fixed with 10% formalin and embedded in paraffin. The tumor sections were stained with hematoxylin-eosin and evaluated by two individual pathologists.

### Statistical analysis

All numerical data were expressed as mean ± SD. A comparison of means among two or more groups was performed using one-way analysis of variance or nonparametric test, and further confirmed by post-hoc analyses with S-N-K or Games-Howell test. All statistical analyses were conducted using SPSS 11.5 software (SPSS, Chicago, IL). Differences with p < 0.05 were considered as significant.

## Results and discussion

### Tumor specific replication and killing effect of Ad.hTERT-E1A-TK

**Figure 1:**
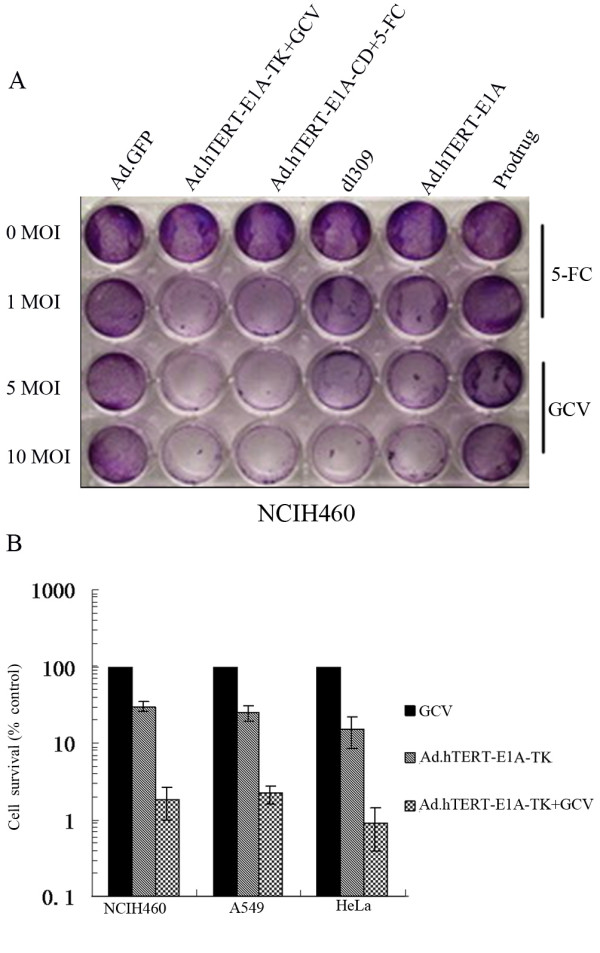
**Tumor cell killing effect of Ad.hTERT-E1A-TK on NSCLC NCIH460 cells**. **A. Crystal violet staining of tumor cells after infection with different adenoviral vectors**. NSCLC NCIH460 cells were plated into 24-well plates and treated with different doses of adenoviral vectors or prodrug or untreated as indicated in figure. 5 days later the plates were stained with crystal violet. **B. CCK-8 assay for surviving cells after infection with Ad.hTERT-E1A-TK**. NSCLC NCIH460 and A549 cells, and cervical carcinoma Hela cellswere plated into 96-well plates and infected by 10 MOI of Ad.hTERT-E1A-TK with or without 0.5 μg/ml GCV. 5 days later the surviving cells were quantified with CCK-8 assay and normalized by untreated cells.

In the present study we generated a novel oncolytic adenoviral vector, Ad. hTERT-E1A-TK, in which tumor selective replication was mediated by the hTERT promoter and HSV-TK gene expression was controlled by CMV promoter. Given Ad.hTERT-E1A-TK contained a suicide gene HSV-TK, we first examined TK expression in Ad.hTERT-E1A-TK infected cells by Western blot. Our results showed that TK expression could be detected in Ad.hTERT-E1A-TK-infected tumor cells but not in control cells (Additional file 2). We next examined Ad.hTERT-E1A-TK/GCV induced cytopathic effect. As shown as crystal violet staining in Fig. 1A and Additional file 3, Ad.hTERT-E1A-TK/GCV was able to kill different type of tumor cells including NCIH460, SW1990, SMMC-7721 and Hela. Its tumor killing effect was comparable with other oncolytic adenoviral vector such as Ad.hTERT-E1A-CD/5-FC, and even superior to Ad.hTERT-E1A as well as wild type adenovirus dl309 in most tested cell lines. Furthermore, Ad.hTERT-E1A-TK killed tumor cell in dose dependent manner. Ad.hTERT-E1A-TK induced tumor cell killing effect was further confirmed by CCK-8 assay. As shown in Fig. 1B, two NSCLC cell lines, NCIH460 and A549, and one human cervical carcinoma cell line Hela showed significant reduction in surviving cells after Ad.hTERT-E1A-TK infection, and GCV could further enhance Ad.hTERT-E1A-TK induced tumor cell killing effect.

**Figure 2:**
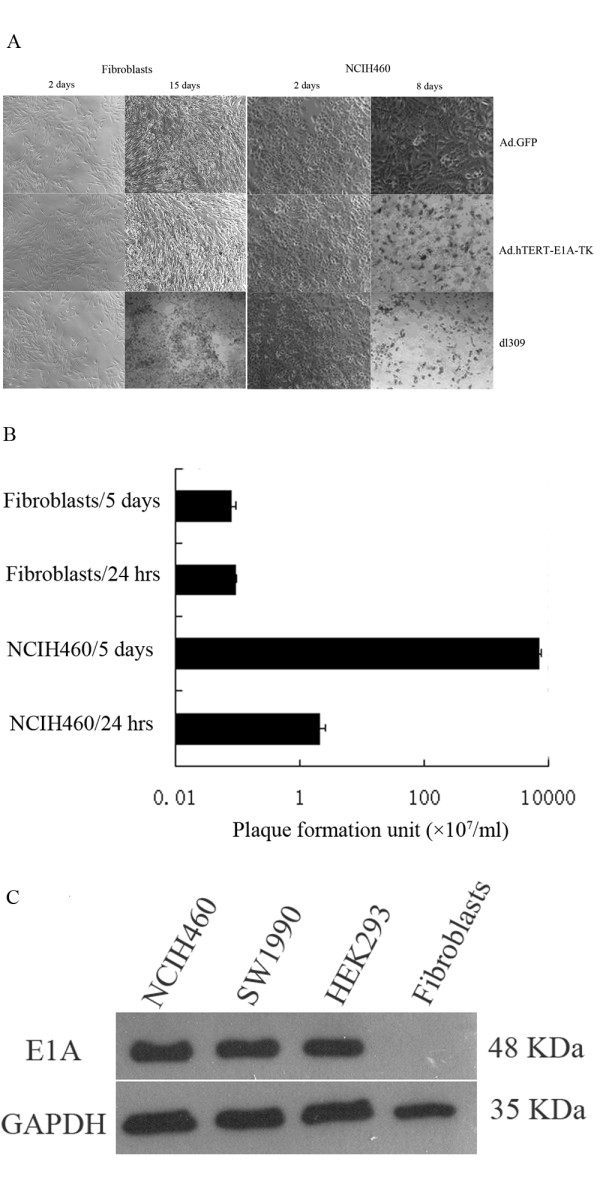
**Selective replication and oncolysis of Ad.hTERT-E1A-TK**. **A. Comparison of cytopathic effects between NSCLC NCIH460 and primary fibroblasts**. NSCLC NCIH460 and primary fibroblasts were plated into 6-well plates and infected with 10 MOI of Ad.hTERT-E1A-TK, dl309 or Ad.GFP. 5 days later cytopathic effects were observed and photographed by light microscopy. **B. The virus progeny production in NCIH460 cells and primary fibroblasts**. NCIH460 and primary fibroblasts were infected with 10 MOI of Ad.hTERT-E1A-TK for 4 h then washed once with PBS and then cultured with fresh medium. On 24 h and day 5 post infection, the cells were harvested for plaque assay. The plaques on HEK293 cells were counted and plotted. **C. Western blotting analysis of E1A gene expression**. NCIH460 and SW1990 Cells were infected with Ad-hTERT-E1A-TK at a MOI of 10. Cell lysates were harvested 48 h later, and immunobloted by anti E1A antibody. HEK293 cells which had been transfected with E1A were used as positive control, and uninfected NCIH460 cells were used as negative control.

In order to demonstrate that Ad.hTERT-E1A-TK induced tumor cell killing effect was tumor specific, we compared the cytopathic effect between NCIH460 tumor cells and primary fibroblasts after 10 MOI of Ad.GFP, Ad.hTERT-E1A-TK or dl309 infection. The non-replicative adenovirus Ad.GFP caused no CPE in either tumor or normal cells, while wild type adenovirus dl309 caused similar CPE in both tumor and normal cells. Interestingly, Ad.hTERT-E1A-TK did not cause CPE in primary fibroblasts but caused CPE in tumor cells which is similar with that in dl309 infected tumor cells (Fig. 2A). In order to confirm that Ad.hTERT-E1A-TK induced tumor specific killing effect was associated with its tumor specific replication, we performed plaque assay to quantify viral progeny production. As shown in Fig. 2B, Ad.hTERT-E1A-TK progenies detected in NCIH460 cells were approximately 7000 times more than that detected in primary fibroblasts. In more detail, about 2 × 10^7 ^and 2 × 10^5 ^of plaques were detected in supernatant from Ad.hTERT-E1A-TK infected NCIH460 cells and primary fibroblasts at 24 h after infection, whereas on day 5 the plaques were 7 × 10^10 ^and 1 × 10^5 ^in supernatant from NCIH460 cells and primary fibroblasts respectively. The plaques detected at 24 h post infection might derive from left vital adenovirus in the infected cells, however, the plaques detected on day 5 faithfully reflected the differential replication between tumor and normal cells.

The expression of E1A gene can also be regarded as an indirect evidence for adenoviral replication, hence we performed Western blot to detect E1A expression in Ad.hTERT-E1A-TK infected NCIH460 cells and primary fibroblasts. 48 h after infection E1A expression was only detected in NCIH460 cells but not in primary fibroblasts which supported Ad.hTERT-E1A-TK selective-replication in tumor cells (Fig. 2C).

### GCV enhanced Ad.hTERT-E1A-TK tumor killing effect in vitro

**Figure 3:**
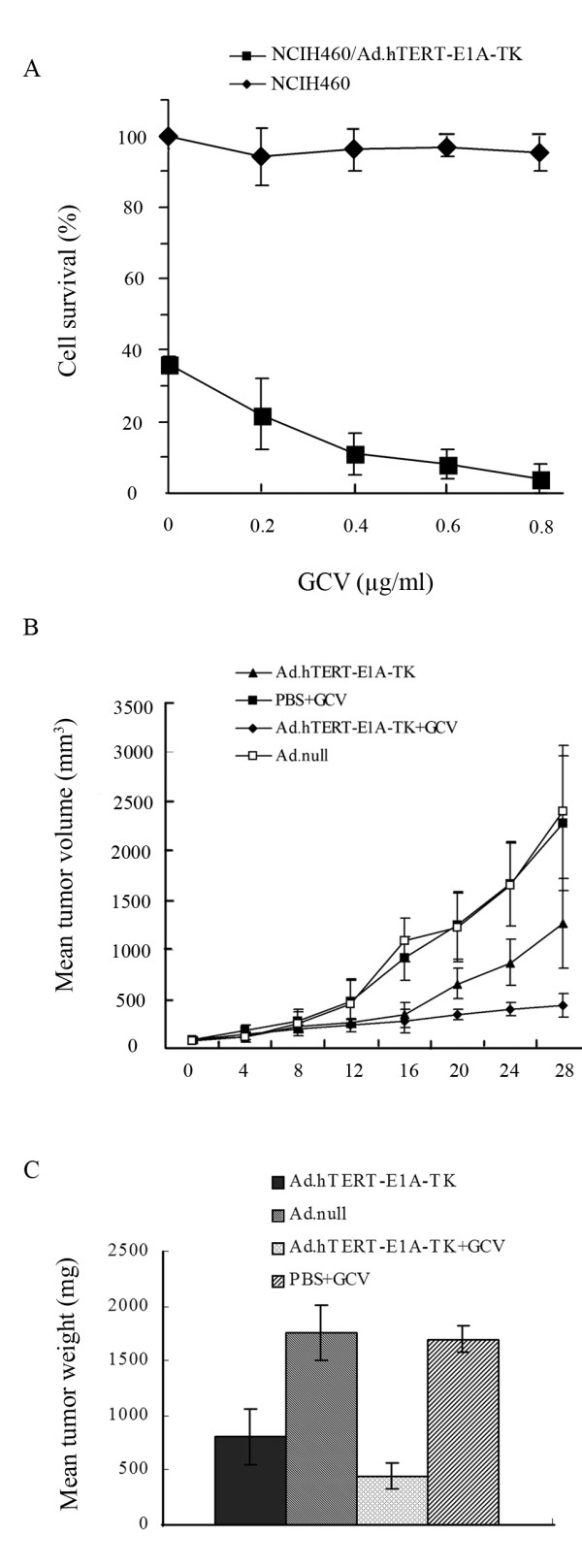
**GCV enhanced inhibition on tumor growth in vitro and in vivo**. **A. GCV enhanced Ad.hTERT-E1A-TK tumor killing effect in vitro**. NCIH460 tumor cells were infected with 10 MOI of Ad.hTERT-E1A-TK and then exposed to different concentration of GCV for 5 days. The surviving cells were quantified with CCK-8 assay and plotted. **B. Ad.hTERT-E1A-TK/GCV suppressed tumor growth in vivo**. NCIH460 xenograft tumors in nude mice were treated by Ad.null, PBS plus GCV, Ad.hTERT-E1A-TK alone or Ad.hTERT-E1A-TK plus GCV. Tumor sizes were measured twice a week using calipers and tumor volumes were plotted. **C. Tumor weight at the end of the study**. On day 28 post treatment, all animals were sacrificed and the tumors were removed and weighted. The data represent the mean ± SD from at least 7 animals per group.

The advantage of using suicide gene as therapeutic gene is that it can convert non-toxic prodrug into toxic therapeutic agent. Since this converting process occurs in tumor site, it will save normal tissues from potential damage by systemic administration of toxic therapeutic agent. Next we investigated whether GCV could enhance Ad.hTERT-E1A-TK mediated tumor cell killing effect in vitro. To do this, NCIH460 tumor cells were infected with 10 MOI of Ad.hTERT-E1A-TK and then exposed to different concentration of GCV for 5 days. According to our previous data, 10 MOI of Ad.GFP infection resulted in approximately 80% GFP positive expression cells in NCIH460 that suggested NCIH460 cells could be efficiently transduced by Ad, therefore, we applied 10 MOI of Ad.hTERT-E1A-TK to NCIH460 cells. The cells, infected by Ad.hTERT-E1A-TK alone for 5 days, showed about 60% death while the addition of GCV resulted in significantly more cell death. For example, about 85% or 95% cell death were observed when GCV was o.4 μg/ml or 0.8 μg/ml respectively. Therefore, GCV synergistically-enhanced Ad.hTERT-E1A-TK induced tumor cell killing effect in dose-dependent manner (Fig. 3A).

### Ad.hTERT-E1A-TK/GCV suppressed tumor growth in vivo

The therapeutic effect of Ad.hTERT-E1A-TK alone or in combination with GCV was evaluated using human NSCLC nude mice models. The mice models were established by subcutaneous injection of NCIH460 cells. When the tumors grew up to approximately 100 mm^3^, about 1 × 10^9 ^PFU of Ad.null orAd.hTERT-E1A-TK in 100 μl PBS or 100 μl PBS alone was injected into tumors respectively. On the 3rd day post virus injection, GCV (100 mg/kg/day) was intraperitoneally administered for 14 consecutive days. The tumor growth was assessed by measuring bi-dimensional diameters twice a week with calipers. As shown in Fig. 3B, the tumors treated by Ad.hTERT-E1A-TK alone or Ad.hTERT-E1A-TK plus GCV started to grow slowly on the 8^th ^day after treatment when compared with that treated with Ad.null or PBS plus GCV. On 16^th ^day, the differences became more significant. At the end of observation, the average tumor sizes were 2440.00 mm^3^, 2287.00 mm^3^, 1274.50 mm^3 ^and 435.01 mm^3 ^in group of Ad.null, PBS plus GCV, Ad.hTERT-E1A-TK alone, and Ad.hTERT-E1A-TK plus GCV, respectively. Since Ad.null and PBS plus GCV showed no difference in tumor growth or tumor size (p > 0.05), we took both Ad.null and PBS plus GCV together as control. The tumor growth curve and tumor size between control and Ad.hTERT-E1A-TK or Ad.hTERT-E1A-TK plus GCV was significantly different (p = 0.025 or p = 0.008) and by 54.39% and 74.34% reduction in tumor weight in Ad.hTERT-E1A-TK or Ad.hTERT-E1A-TK plus GCV treated groups compared with controls. More importantly, the tumor growth curve and tumor size between Ad.hTERT-E1A-TK and Ad.hTERT-E1A-TK plus GCV also showed different (p = 0.040), it was about 43.75% reduction in tumor size in Ad.hTERT-E1A-TK plus GCV treated group (Fig. 3C).

It is necessary to mention that the timing for prodrug giving was on the 3^rd ^day in this study. The reason was mainly dependent on our previous study in which the transgene expression reached the peak on the 3^rd ^day after intratumoral injection of either replication-competent or replication-deficient adenoviral vectors. In that study the transgene (red fluorescent protein, RFP) expression was visualized by in vivo imaging (data not shown), it reached the peak on the 3rd day which might reflect full replication and distribution of adenoviral vectors in tumor tissue. Whether the advanced administration of GCV would result in the suppression on adenoviral replication in tumor tissues or interferer with therapeutic efficacy of Ad.hTERT-E1A-TK has not been investigated in this study.

**Figure 4:**
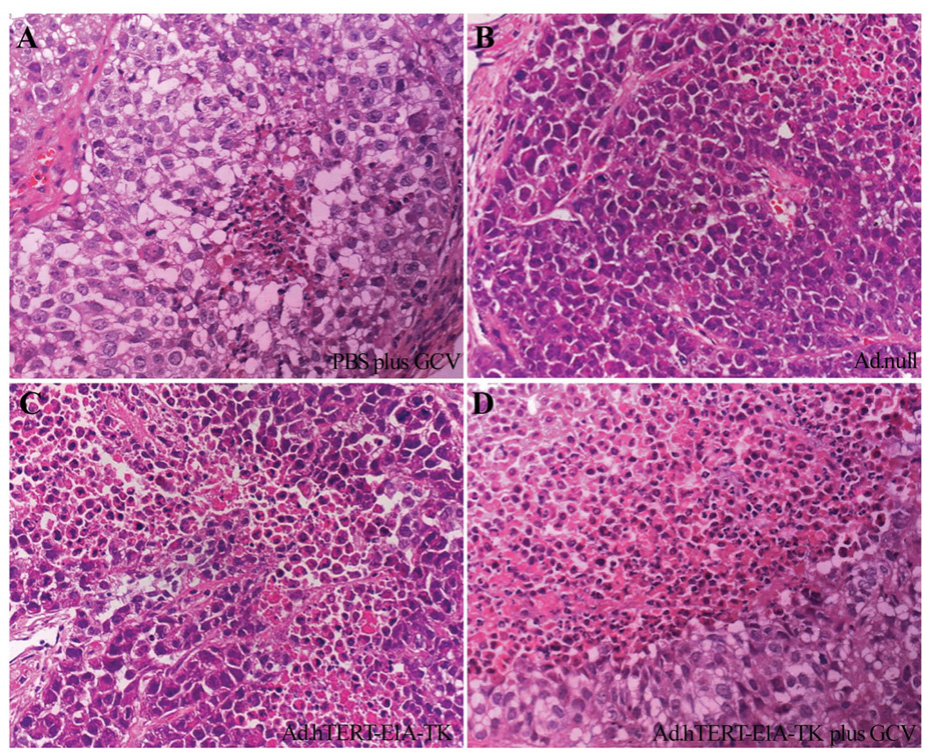
**Histological examinations of NCIH460 tumors treated by different conditions**. The hematoxylin-eosin stain of NCIH460 tumors treated by different conditions, PBS plus GCV treated (**A**); Ad.null treated (**B**); Ad.hTERT-E1A-TK alone treated (**C**). Ad.hTERT-E1A-TK plus GCV treated (**D**). The necrosis is barely seen in control groups (**A **and **B**), while there are obvious necrotic areas and numerous apoptotic bodies in the tumor tissues treated by Ad.hTERT-E1A-TK alone or Ad.hTERT-E1A-TK plus GCV treated (**C **and **D)**. Original magnification ×400.

The tumor sections from different groups also showed differential histopathologic features. The most obvious difference was the numbers of apoptotic cells and the range of necrosis area. As shown in Fig. 4, tumors treated with Ad.hTERT-E1A-TK plus GCV showed wider necrosis and more dark-stained and condensed nuclei.

## Conclusion

Taking together, we have generated a novel oncolytic adenoviral vector in which the main difference with currently used oncolytic adenoviral vector ONYX-015 is hTERT controlled replication and armed with HSV-TK. The hTERT promoter used in this study is high stringency and provide the base for tumor-specific replication. Ad.hTERT-E1A-TK itself was able to inhibit tumor growth thanks to its replicative ability and oncolytic effect. Moreover, its tumor killing effect could be further enhanced by prodrug GCV. Our study showed that Ad.hTERT-E1A-TK/GCV could efficiently kill NSCLC tumor cells both in vitro and in vivo. Therefore, we concluded that Ad.hTERT-E1A-TK, as a potent and safe antitumor strategy, could provide a potential new option for NSCLC biotherapy.

## Abbreviations

NSCLC: Non-small cell lung cancer; HSV: Herpes Simplex Virus; TK: Thymidine Kinase; CGV: gancyclovir; hTERT: human telomerase reverse transcriptase; MOI: multiplicity of infection; PFU: plaque forming units; CPE: cytopathic effect; CCK-8: Cell Counting Kit-8.

## Competing interests

The authors declare that they have no competing interests.

## Authors' contributions

JFZ carried out most of the experiments and organized data for manuscript. FW, HPW, HML, XFC performed some experiments involving in viral construction, package, Western blot or cell culture. WQ and PKR participated in data organization and manuscript drafting. QH performed project design and manuscript writing. All authors read and approved the final manuscript.

## Supplementary Material

Additional file 1 **Schematic diagram of Ad.hTERT-E1A-CD or Ad.hTERT-E1A-TK adenoviral construct**. Ad.hTERT-E1A-CD or Ad.hTERT-E1A-TK adenoviral vector had been constructed in the way described in this figure. ITR, inverted repeats of the adenovirus genome; ΔE1 and ΔE3, E1 and E3 region deleted. Click here for file

Additional file 2 **Western blotting analysis of TK gene expression**. NCIH460 Cells were infected with Ad-hTERT-E1A-TK at a MOI of 10. Cell lysates were harvested 48 h later, and immunobloted by anti HA-tag antibody. NCIH460 Cells which had been transfected with plasmid containing TK gene were used as positive control, and uninfected NCIH460 cells were used as negative control. Click here for file

Additional file 3 **Tumor cell killing effect of Ad.hTERT-E1A-TK on different tumor cells**. Crystal violet staining of tumor cells after infection with different adenoviral vectors. SW1990, SMMC-7721 and HeLa cells were plated into 24-well plates and treated with different dose of adenoviral vectors or prodrug or untreated as indicated in figure. 5 days later the plates were stained with crystal violet. Click here for file
