# Supplementary figures and images for: Potent anti-tumor activity of telomerase-dependent and HSV-TK armed oncolytic adenovirus for non-small cell lung cancer in vitro and in vivo
Source: J Exp Clin Cancer Res. 2010 May 20;29(1):52. doi: 10.1186/1756-9966-29-52 (PMC2890545; doi:10.1186/1756-9966-29-52)

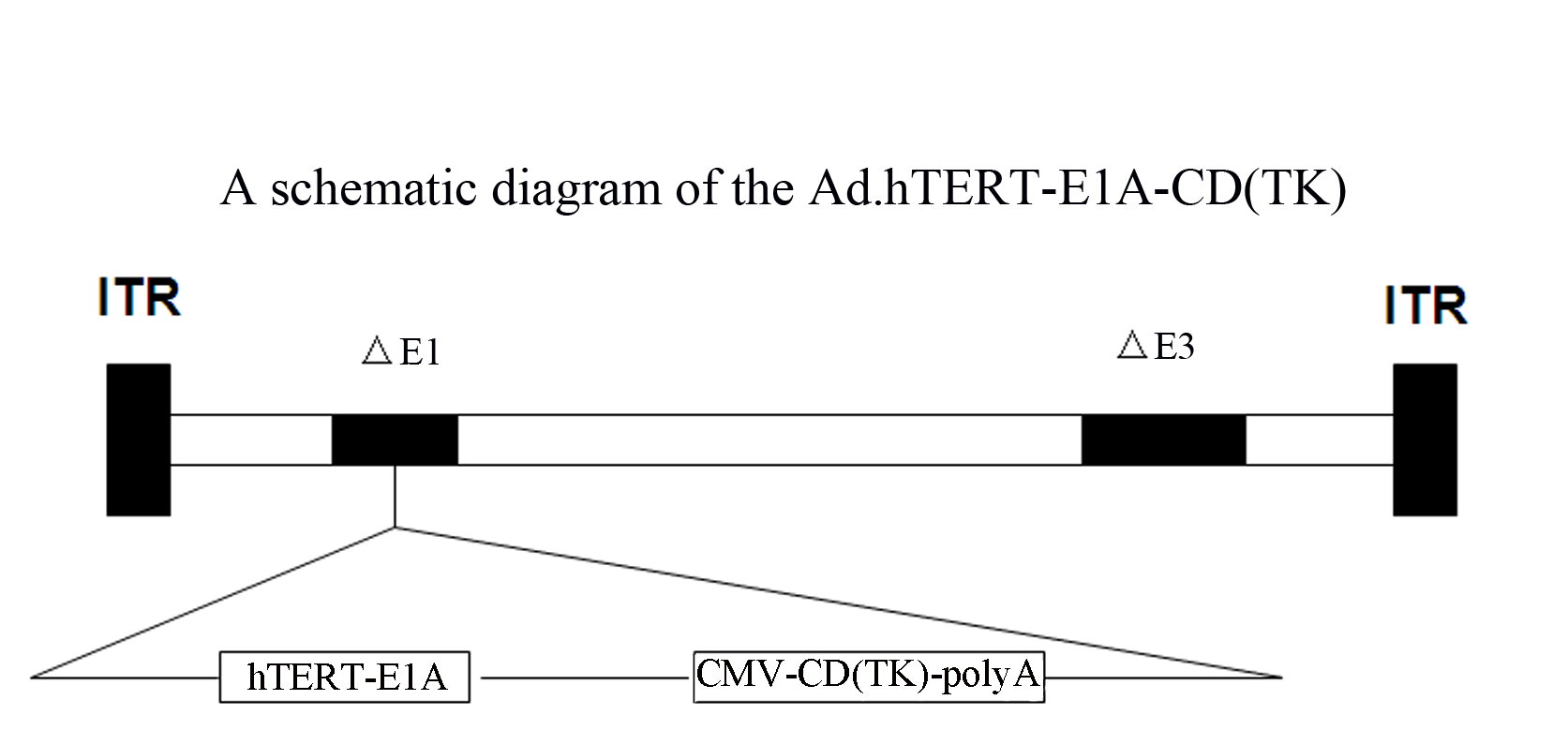

Supplement: Additional file 1 — Schematic diagram of Ad.hTERT-E1A-CD or Ad.hTERT-E1A-TK adenoviral construct. Ad.hTERT-E1A-CD or Ad.hTERT-E1A-TK adenoviral vector had been constructed in the way described in this figure. ITR, inverted repeats of the adenovirus genome; ΔE1 and ΔE3, E1 and E3 region deleted. [file 1756-9966-29-52-S1.TIFF]

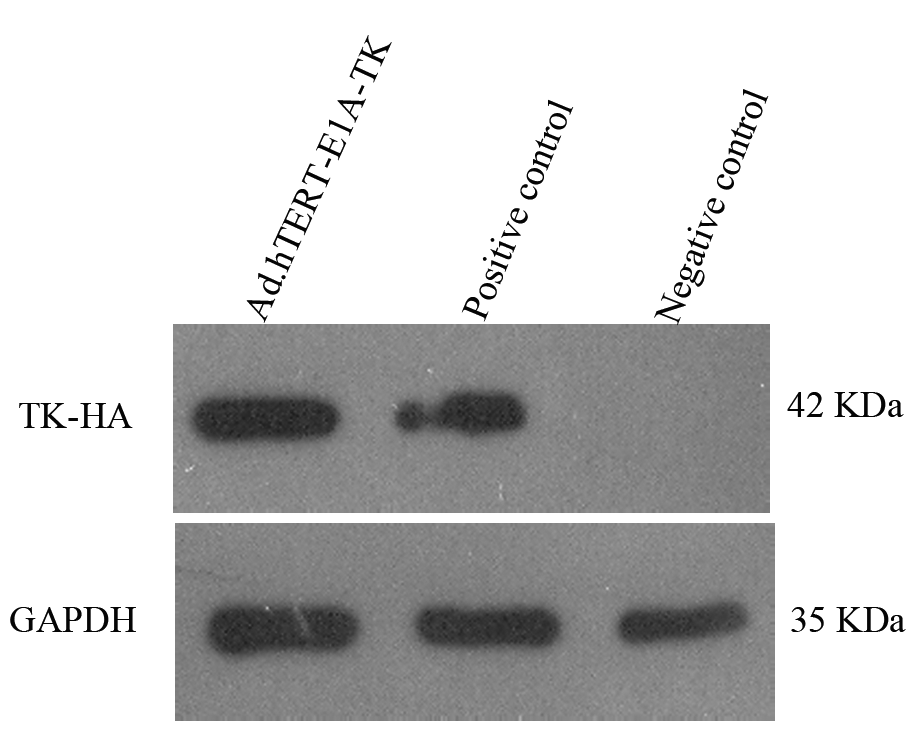

Supplement: Additional file 2 — Western blotting analysis of TK gene expression. NCIH460 Cells were infected with Ad-hTERT-E1A-TK at a MOI of 10. Cell lysates were harvested 48 h later, and immunobloted by anti HA-tag antibody. NCIH460 Cells which had been transfected with plasmid containing TK gene were used as positive control, and uninfected NCIH460 cells were used as negative control. [file 1756-9966-29-52-S2.TIFF]

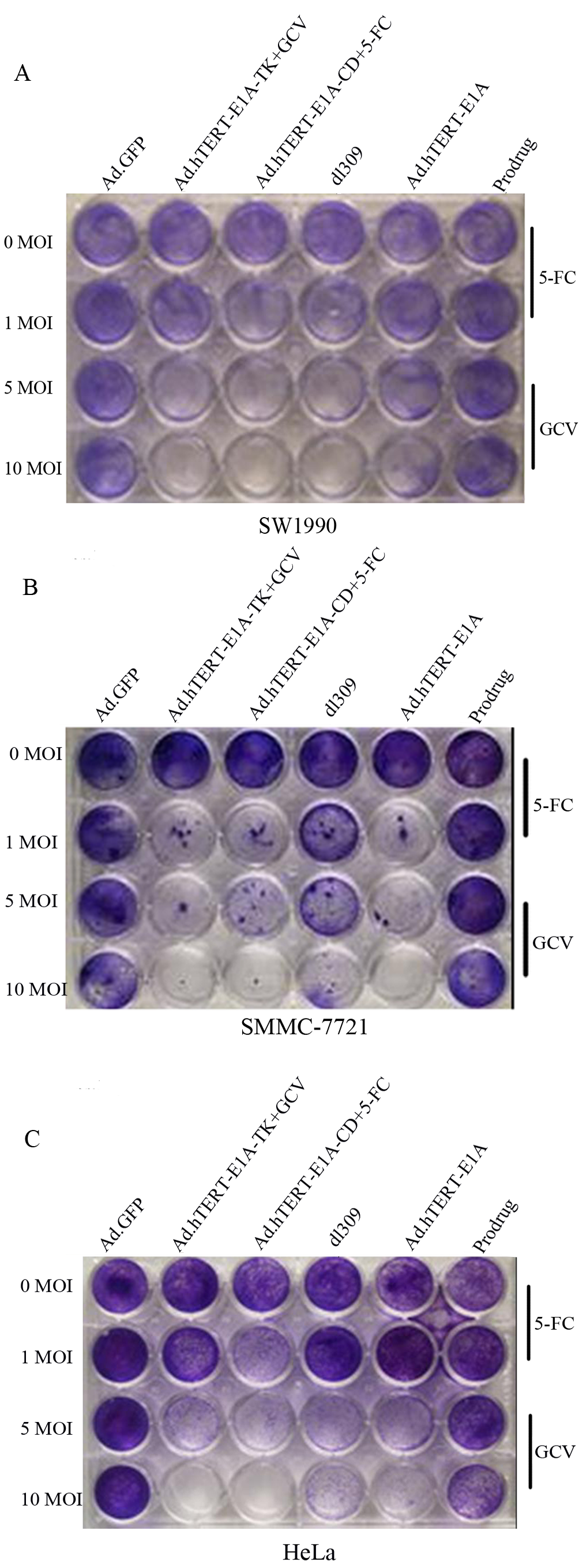

Supplement: Additional file 3 — Tumor cell killing effect of Ad.hTERT-E1A-TK on different tumor cells. Crystal violet staining of tumor cells after infection with different adenoviral vectors. SW1990, SMMC-7721 and HeLa cells were plated into 24-well plates and treated with different dose of adenoviral vectors or prodrug or untreated as indicated in figure. 5 days later the plates were stained with crystal violet. [file 1756-9966-29-52-S3.TIFF]
